# Metabolic signatures in the conversion from gestational diabetes mellitus to postpartum abnormal glucose metabolism: a pilot study in Asian women

**DOI:** 10.1038/s41598-021-95903-w

**Published:** 2021-08-12

**Authors:** Xi-Meng Wang, Yan Gao, Johan G. Eriksson, Weiqing Chen, Yap Seng Chong, Kok Hian Tan, Cuilin Zhang, Lei Zhou, Ling-Jun Li

**Affiliations:** 1 Singapore Institute for Clinical Sciences, Agency for Science Technology and Research (A*STAR), Singapore, Singapore; 2 Department of Epidemiology, Guangdong Cardiovascular Institute, Guangdong Provincial People’s Hospital, Guangdong Academy of Medical Sciences, Guangzhou, Guangdong China; 3 Singapore Eye Research Institute, Singapore National Eye Centre, Singapore, Singapore; 4 Department of Obstetrics and Gynecology, Yong Loo Lin School of Medicine, National University of Singapore, Singapore, Singapore; 5 Unit of General Practice and Primary Health Care, University of Helsinki, Tukholmankatu 8 B, P.O. Box 20, 00014 Helsinki, Finland; 6 Department of Epidemiology, School of Public Health, Sun-Yat Sen University, Guangzhou, Guangdong China; 7 Division of Obstetrics and Gynecology, KK Women’s and Children’s Hospital, Singapore, Singapore; 8 Obstetrics and Gynecology Academic Clinical Programme, Duke-NUS Graduate Medical School, Singapore, Singapore; 9 Epidemiology Branch, Division of Intramural Population Health Research, Eunice Kennedy Shriver National Institute of Child Health and Human Development, National Institutes of Health, Bethesda, MD USA; 10 Department of Ophthalmology, Yong Loo Lin School of Medicine, National University of Singapore, Singapore, Singapore; 11 Ophthalmology and Visual Sciences Academic Clinical Research Program, Duke-NUS Medical School, National University of Singapore, Singapore, Singapore

**Keywords:** Gestational diabetes, Type 2 diabetes, Predictive markers

## Abstract

We aimed to identify serum metabolites related to abnormal glucose metabolism (AGM) among women with gestational diabetes mellitus (GDM). The study recruited 50 women diagnosed with GDM during mid-late pregnancy and 50 non-GDM matchees in a Singapore birth cohort. At the 5-year post-partum follow-up, we applied an untargeted approach to investigate the profiles of serum metabolites among all participants. We first employed OPLS-DA and logistic regression to discriminate women with and without follow-up AGM, and then applied area under the curve (AUC) to assess the incremental indicative value of metabolic signatures on AGM. We identified 23 candidate metabolites that were associated with postpartum AGM among all participants. We then narrowed down to five metabolites [*p-cresol* sulfate, linoleic acid, glycocholic acid, lysoPC(16:1) and lysoPC(20:3)] specifically associating with both GDM and postpartum AGM. The combined metabolites in addition to traditional risks showed a higher indicative value in AUC (0.92–0.94 vs. 0.74 of traditional risks and 0.77 of baseline diagnostic biomarkers) and R^2^ (0.67–0.70 vs. 0.25 of traditional risks and 0.32 of baseline diagnostic biomarkers) in terms of AGM indication, compared with the traditional risks model and traditional risks and diagnostic biomarkers combined model. These metabolic signatures significantly increased the AUC value of AGM indication in addition to traditional risks, and might shed light on the pathophysiology underlying the transition from GDM to AGM.

## Introduction

Gestational diabetes mellitus (GDM) is a hyperglycemic condition first recognized during pregnancy^1^. GDM affects 4.5% to 20.3% of pregnancies among Asian women depending on different countries and GDM diagnostic criteria^2^. GDM increases the woman’s lifetime risk of developing abnormal glucose metabolism (AGM), including prediabetes and type 2 diabetes (T2D)^3,4^. Additionally, these women are also at higher risk of developing cardiovascular disease and renal disease^5,6^. Therefore, identifying more convenient approaches such as novel biomarkers to quantify the risk of AGM could be beneficial for risk stratification and early postpartum intervention among women with GDM.

It is well known that metabolites are the end products of specific cellular regulatory processes. The levels of metabolites could reflect the ultimate response of biological systems to genetic or environmental changes^7^. In terms of the transition from GDM to postpartum AGM, the capture of metabolic signals underlying postpartum AGM might be indicative and even predict this process. Emerging studies demonstrated that specific metabolomic biomarkers improved the prediction of the transition risk from GDM to T2D, including lipids [i.e., Cholesteryl ester (20:4), Lphosphatidylethanolamine (36:2), Phosphatidylserine (38:4), Lphosphatidylserine (C40:5)] and amino acids (i.e., branched-chain amino acids, Hexose)^8–11^. However, there is still a lack of adequate understanding in this field of research. Firstly, previous studies have only focused on metabolic biomarkers in the transition from GDM to T2D, mainly ignoring the transition from GDM to prediabetes. Secondly, these prior studies have primarily used targeted approaches to assess metabolic profiles and tested specific categories of metabolites (e.g., lipids) based on potential mechanisms and interests^8–10^. Such methods limit the consideration of the full spectrum of human metabolic profiles and misinterpret the underlying mechanisms between GDM and the development of AGM with selection bias in specific categories of metabolites. Thirdly, these existing studies mainly focused on non-Asian populations in the US and Australia while lacking data on the Asian population, which are at a high risk of both GDM and T2D^12^. Lastly, all the present studies reported metabolic biomarkers identified only in GDM women. However, there is also a trend in developing postpartum AGM among women without a history of GDM^13^.

In this pilot with 100 women nested in a Singapore birth cohort, we identified metabolic signatures associated with postpartum AGM and GDM via an untargeted and discovery-based metabolomic approach. Subsequently, we investigated the postpartum AGM indicative value given by such metabolic signatures, in addition to traditional risks including a family history of T2D and body mass index (BMI).

## Results

Of the 100 participants, 24 out of 50 women with GDM (46%) and 17 out of 50 women without GDM (34%) developed AGM after 5 years’ follow-up (*p* = 0.22). Women with GDM at baseline had similar pre-pregnancy BMI (22.8 vs. 21.6, *p* = 0.54), lower gestational weight gain at 26–28 weeks of gestation (8.2 vs. 8.8, *p* = 0.07), and lower higher BMI at follow-up (24.1 vs. 25.9, *p* = 0.54) compared with women without GDM at baseline (Table 1).

**Table 1:** Sociodemographic and clinical characteristics at baseline and 5-year postpartum follow-up of the study population.

Maternal characteristics	GDM subjects (N = 50)	Non-GDM subjects (N = 50)	*p*-value
	Median (IQR) or N (%)	Median (IQR) or N (%)	
**At baseline (24–28 weeks of gestation)**			
Age, years	34.0 (6.0)	33.0 (9.0)	0.40^†^
Ethnicity			
Chinese	25 (50%)	30 (61%)	0.90^‡^
Indian	8 (16%)	12 (24%)	
Malay	17 (34%)	8 (16%)	
College degree, yes	23 (46%)	22 (44%)	0.85^‡^
Family history of T2D, yes	22 (44%)	23 (46%)	0.85^‡^
nulliparous, yes	24 (48%)	16 (32%)	0.10^‡^
Pre-pregnancy BMI, kg/m^2^	22.8 (6.0)	21.6 (6.5)	0.94^†^
26–28 weeks GWG, kg	8.2 (5.8)	8.8 (7.0)	0.06^†^
**At 5-year postpartum follow-up**			
Cumulative numbers of GDM episodes			
None	0 (0%)	42 (84%)	< 0.01^‡^
1	40 (80%)	6 (12%)	
2 or more	10 (20%)	2 (4%)	
Age at year 5, years	39.0 (7.0)	38.0 (9.0)	0.40^†^
BMI at year 5	24.1 (5.9)	25.9 (7.3)	0.54^†^
Baseline-year 5 BMI increase	1.2 (2.5)	2.0 (4.2)	0.31^†^
Number of pregnancies during follow-up	1.0 (1.0)	1.5 (1.0)	0.27^†^
Glucose metabolism at year 5			
Normal glucose metabolism	26 (52%)	33 (66%)	0.19^‡^
IFG/IGT	22 (44%)	15 (30%)	
T2D	2 (4%)	2 (4%)	

*AGM* abnormal glucose metabolism, *T2D* type 2 diabetes, *BMI* body mass index, *GWG* gestational weight gain, *GDM* gestational diabetes mellitus, *IFG* impaired fasting glucose, *IGT* impaired glucose tolerance, *IQR* interquartile range.

^†^Generalized linear mixed model with matching factors (baseline age and/or pre-pregnancy BMI when appropriate) modeled as random intercepts.

^‡^Generalized estimating equations accounting for matched pairs.

**Figure 1:**
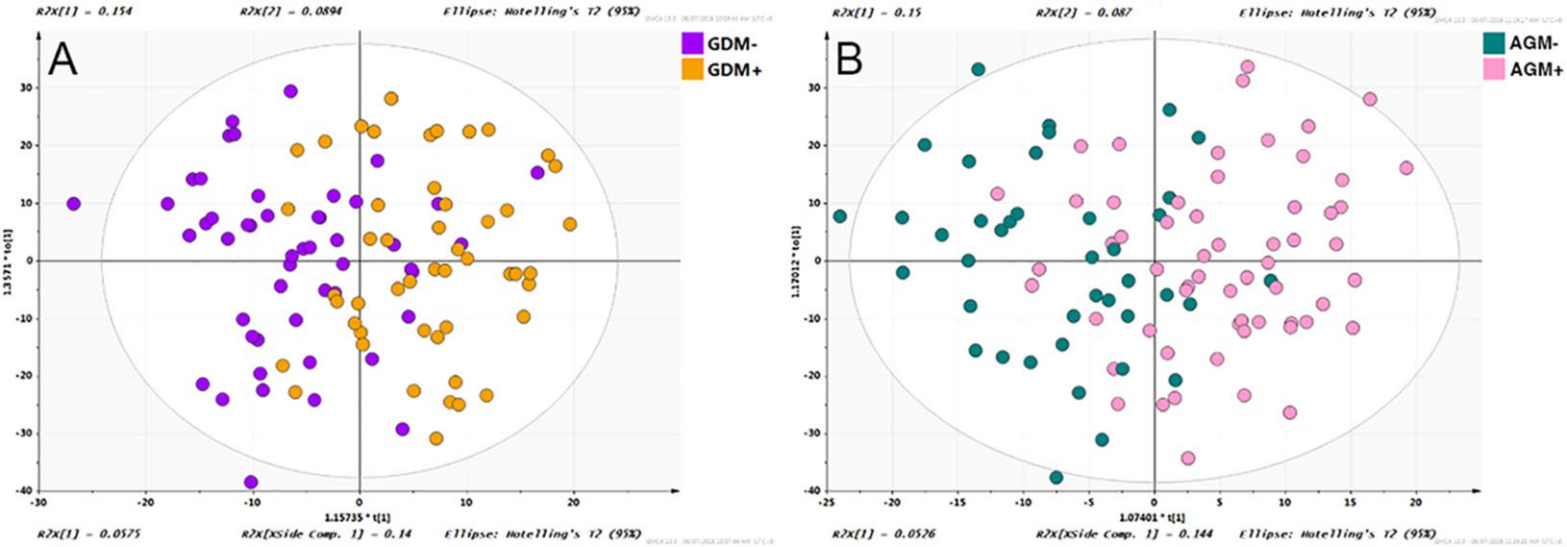
OPLS-DA score plots of metabolic profiles of gestational diabetes mellitus/normal glucose metabolism women at baseline (24–28 weeks of gestation), and abnormal glucose metabolism (AGM)/normal glucose metabolism women at 5-year’s follow-up. (**A**) The purple dots and the yellow dots represent women with and without GDM at baseline, respectively; (**B**) The pink dots and the green dots represent women with and without AGM at 5-year’s follow-up, respectively.

Figure 1 illustrate women's metabolic profiles with and without AGM at the 5-year follow-up in OPLS-DA score plots. A total of 31 serum metabolites were found at different levels between women with and without 5-year postpartum AGM in a crude model (OPLS-DA VIP score > 1, Mann–Whitney U test or t-test *p*-values 0.0006 to 0.049). After adjusting for age at follow-up, race/ethnicity, BMI at follow-up, college education, family history of T2D and nulliparity, 23 metabolites remained statistically significant in association with 5-year postpartum AGM (FDR < 0.1), of which 5 metabolites were able to further differentiate women with a history of GDM (Table 2). Regression coefficients of all 23 metabolites are presented in Supplementary Table 1.

**Table 2:** Metabolites that were associated with 5-year postpartum abnormal glucose metabolism after multiple adjustment.

Metabolites ID	Chemical name	Association with 5-year postpartum AGM^†^			Potential predictor of AGM among GDM women
		regardless GDM status	GDM subjects	Non-GDM subjects	
187.0077_3.20_rn	*p-Cresol* sulfate	+	+	–	●
212.0026_2.41_rn	Indoxyl sulfate	+	–	–	
217.0177_3.95_rn	3-CMPFP	+	–	–	
254.9827_0.42_rn	Ascorbic acid-2-sulfate	+	–	–	
271.2053_6.85_rp	Alpha-Amylcinnamyl isovalerate	+	–	–	
274.1047_0.42_rn	Glutaminylglutamic acid	+	–	+	
279.2316_0.44_hp	Alpha-Linolenic acid	+	–	–	
279.2323_9.57_rp	Alpha-Linolenic acid	+	–	–	
281.2476_0.44_hp	Linoleic acid	+	+	–	●
351.1640_4.20_rn	Epiandrosterone sulfate	+	–	–	
367.1062_0.46_rn	3-O-Feruloylquinic acid	+	–	–	
369.1742_5.47_rn	Epiandrosterone sulfate	+	–	–	
369.1746_4.93_rn	Epiandrosterone sulfate	+	–	–	
378.1011_5.22_rn	Chondroitin	+	–	–	
429.3001_8.36_rp	Spirotaccagenin	+	–	+	
446.2915_5.37_rn	Glycocholic acid	–	+	–	●
449.1307_4.15_rn	Oleoside 11-methyl ester	+	–	–	
464.3024_4.78_rn	LysoPC(16:1)	+	–	–	
471.2434_4.21_rn	LysoPE(20:4)	+	–	–	
494.3249_8.41_rp	LysoPC(16:1)	–	+	–	●
546.3563_8.78_rp	LysoPC(20:3)	+	+	–	●
559.4713_0.44_hp	DG(18:4)	+	–	–	
577.4823_0.44_hp	DG(18:3)	+	–	–	

*FDR* false discovery rate.

^†^All models were adjusted for age at year 5, ethnicity (Chinese as reference), college education, BMI at year 5, family history of T2D, and nulliparity.

+ : Associated with 5-year postpartum AGM with FDR < 0.1, −: FDR ≥ 0.1, ●: considered as potential indicator of AGM among GDM women.

After further controlling for collinearity with ridge regression, all 5 metabolites remained significant in relation to 5-year postpartum AGM (*p*: 0.001 to 0.018) (Supplementary Table 2). These five metabolites, namely *p-cresol* sulfate, linoleic acid, glycocholic acid, lysophosphatidylcholines [LysoPC(16:1) and LysoPC(20:3)], were included in the AGM indication models. Higher serum levels of LysoPC(16:1) and LysoPC(20:3) were associated with increased risk of 5-year postpartum AGM, while higher levels of *p-cresol* sulfate, linoleic acid, and glycocholic acid were associated with reduced risk of 5-year postpartum AGM. LysoPC(16:1) and LysoPC(20:3) were analyzed separately to avoid biological collinearity.

**Figure 2:**
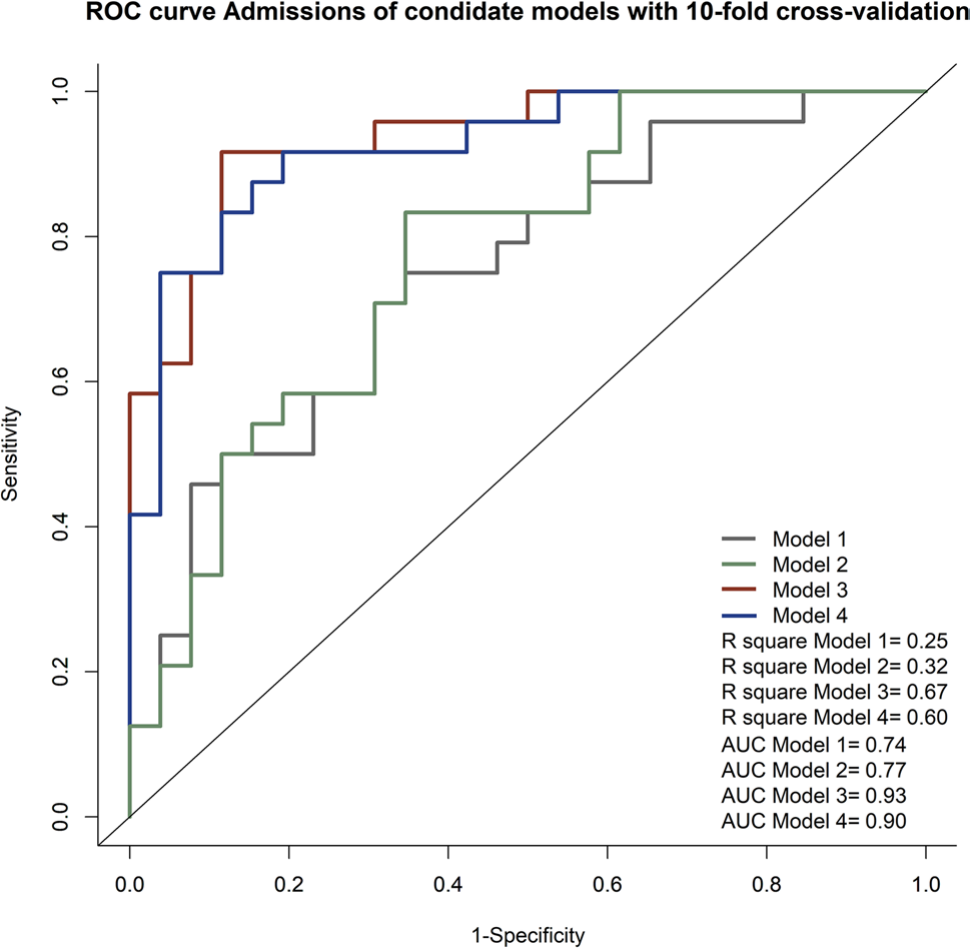
Receiver operating characteristic (ROC) curve admissions of the indicative models on AGM at 5-year’s follow-up. The gray line represents the ROC curve of Model 1: AGM at year 5 ~ Age at year 5 + Ethnicity + BMI at year 5 + Family History of T2D + Number of GDM Episodes, R^2^ = 0.25, AUC = 0.74; The green line represents the ROC curve of Model 2: AGM at year 5 ~ Age at year 5 + Ethnicity + BMI at year 5 + Family History of T2D + Number of GDM Episodes + Fasting and 2-h glycemic levels at study entry, R^2^ = 0.32, AUC = 0.77; The orange line represents the ROC curve of Model 3: AGM at year 5 ~ *p-cresol* sulfate + linoleic acid + Glycocholic acid + LysoPC(16:1) + Age at year 5 + Ethnicity + BMI at year 5 + Family History of T2D + Number of GDM Episodes + Fasting and 2-h glycemic levels at study entry, R^2^ = 0.70, AUC = 0.94; The blue line represents the ROC curve of Model 4: AGM at year 5 ~ *p-cresol* sulfate + linoleic acid + Glycocholic acid + LysoPC(20:3) + Age at year 5 + Ethnicity + BMI at year 5 + Family History of T2D + Number of GDM Episodes + Fasting and 2-h glycemic levels at study entry, R^2^ = 0.67, AUC = 0.92.

Figure 2 presents the results of the comparison across four models, namely Model 1 (traditional risks), Model 2 (traditional risks and diagnostic biomarkers including fasting and 2-h glycemic levels at study entry), Model 3 [Model 2 and metabolites *p-cresol* sulfate, linoleic acid, glycocholic acid and lysoPC(16:1)], and Model 4 [Model 2 and metabolites *p-cresol* sulfate, linoleic acid, glycocholic acid and lysoPC(20:3)]. Variables included in each model and AUC and R square for each model are presented in Table 3. The AUC (R^2^) for all models were listed accordingly: 0.74 (0.25) in Model 1, 0.77 (0.32) in Model 2, 0.94 (0.70) in Model 3 and 0.92 (0.67) in Model 4. The AUC of Models 3 and 4 were both significantly higher than Model 1 and Model 2 individually and Model 3 yielded the highest indicative value among all stepwise models (Supplementary Table 3).

**Table 3:** Contribution of variables in each regression model for abnormal glucose metabolism.

Model performance	Model 1		Model 2			Model 3		Model 4	
**AUC**^**†**^	0.74		0.77			0. 94		0.92	
**AUC *p*-value**^**‡**^	n/a		0.57 (vs. Model 1)			0.040 (vs. Model 1) 0.038 (vs. Model 2)		0.047 (vs. Model 1) 0.045 (vs. Model 2)	
**R**^**2**^	0.25		0.32			0.70		0.67	
**Variables in models**	*β* (95%CI)	*p*-value	*β* (95%CI)		*p*-value	*β* (95%CI)	*p*-value	*β* (95%CI)	*p*-value
Age at 5-year Follow-up, years	0.21 (0.02, 0.46)	0.05	0.22 (0.01, 0.49)		0.07	0.25 (− 0.08, 0.72)	0.2	0.32 (− 51.39, 4.31)	0.16
**Ethnicity**									
Chinese	Reference	–	Reference		–	Reference	–	Reference	–
Malay	1.94 (− 0.39, 4.61)	0.12	1.85 (− 0.59, 4.62)		0.15	0.84 (− 2.82, 5.07)	0.65	0.29 (− 0.98, 5.41)	0.88
Indian	0.68 (− 1.19, 2.62)	0.47	0.54 (− 1.37, 2.05)		0.58	− 0.84 (− 4.09, 2.47)	0.60	− 1.69 (− 3.53, 4.47)	0.32
BMI at 5-year Follow-up, kg/m^2^	0.14 (− 0.03, 0.33)	0.11	0.20 (0.01, 0.41)		0.05	0.05 (− 0.25, 0.39)	0.74	0.23 (− 0.04, 0.85)	0.21
Family history of T2D, yes	− 0.12 (− 1.8, 1.47)	0.88	− 0.27 (− 2.07, 1.42)		0.76	1.81 (− 1.08, 5.26)	0.25	2.33 (− 0.09, 0.63)	0.15
Cumulative GDM episodes	1.07 (− 0.38, 2.87)	0.19	1.31 (− 0.23, 3.24)		0.13	0.45 (− 1.93, 3.25)	0.73	1.71 (− 0.57, 6.10)	0.29
Baseline fasting glucose	n/a		0.15 (− 1.77, 1.45)		0.85	− 0.32 (− 3.23, 2.27)	0.81	− 1.81 (− 5.41, 1.62)	0.24
Baseline 2 h OGTT glucose	n/a		0.56 (− 0.18, 1.36)		0.14	0.46 (− 0.50, 1.69)	0.38	0.84 (− 5.32, 0.97)	0.19
*p-cresol* sulfate	n/a		n/a			− 0.30 (− 0.71, 0.03)	0.10	− 0.31 (− 0.23, 2.40)	0.12
Linoleic acid	n/a		n/a			− 8.89 (− 18.7, − 1.68)	0.04	− 11.14 (− 0.77, 0.05)	0.02
Glycocholic acid	n/a		n/a			− 13.03 (− 34.35, 1.54)	0.16	− 9.19 (− 22.92, − 2.91)	0.28
LysoPC(16:1)	n/a		n/a			1.75 (0.50, 3.53)	0.02	n/a	
LysoPC(20:3)	n/a		n/a			n/a		1.69 (− 8.40, 4.93)	0.03
(Intercept)	− 13.58 (− 27.2, − 3.02)	0.03				− 13.65 (− 41.15, 7.54)	0.25	− 18.65 (0.03, 0.98)	0.18

*AUC* area under the curve, *BMI* body mass index, *T2D* type 2 diabetes, *GDM* gestational diabetes mellitus.

^†^All models were fitted with tenfold cross validation.

^‡^Z-test against Model 1.

**Figure 3:**
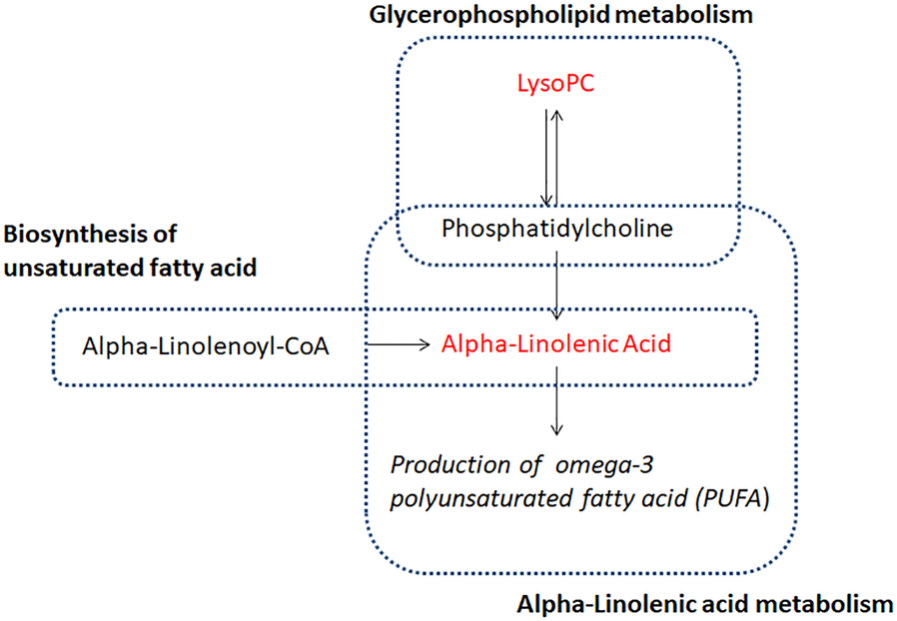
The metabolic network of identified alpha-linolenic acid metabolism.

Additional KEGG pathway analyses with the 31 metabolites that passed OPLS-DA and univariate analysis (Table 4) showed that among all women, AGM-related metabolites were associated with 3 biological pathways with *p*-value < 0.1. They included alpha-linolenic acid metabolism (alpha-linolenic acid) (*p* = 0.03), glycerophospholipid metabolism [LysoPC(16:1) and LysoPC(20:3)] (*p* = 0.09) and biosynthesis of unsaturated fatty acids (alpha-linolenic acid) (*p* = 0.09). The metabolic map that shows the location of alpha-linolenic acid metabolism is presented in Fig. 3.

**Table 4:** Kyoto encyclopedia of genes and genomes (KEGG) pathways of AGM-associated metabolites among all subjects.

KEGG pathway (pathway ID)	Expected	*p*-value^†^	− log(*p*)	Impact	Total intermediates in the pathway	Intermediates associated with AGM (chemical name)
Alpha-Linolenic acid metabolism (hsa00592)	0.034	0.033	3.406	0.333	13	279.2316_0.44_hp (Alpha-Linolenic acid)
Glycerophospholipid metabolism (hsa00564)	0.093	0.090	2.410	0.003	36	494.3249_8.41_rp [LysoPC(16:1)] 546.3563_8.78_rp [LysoPC(20:3)]
Biosynthesis of unsaturated fatty acids (hsa01040)	0.093	0.090	2.410	0.017	36	279.2316_0.44_hp (Alpha-Linolenic acid)

^†^Pathways with *p*-value < 0.1 were included in the results.

## Discussion

Our study identified 5 metabolic signatures [*p-cresol* sulfate, linoleic acid, glycocholic acid, LysoPC(16:1), and LysoPC(20:3)] that were associated with postpartum AGM specifically among women with a history of GDM. In addition to the indicative value of postpartum AGM using traditional risk factors including BMI and family history of T2D as well as glucose level at index pregnancy, these metabolites significantly increased the AUC of the regression model by ~ 20%. Furthermore, our pathway analysis showed that such identified metabolites were involved in either lipid or insulin metabolism.

Emerging evidence has suggested a plausible role of metabolites underlying the transition from GDM to manifest T2D. Several metabolites have been suggested to be predictive of T2D development among women with a history of GDM. These metabolites include branched-chain amino acids, acylcarnitines, fatty acids (i.e., linoleic acid, phospholipids including lysoPCs), and sphingomyelins (i.e., SM (OH) C14:1)^8–11^. However, most existing studies identified these metabolites using targeted approaches focusing on lipids and amino acids^8,9^. Therefore, they may neglect metabolites in other pathways that could significantly contribute to the transition from GDM to T2D.

In our study, serum metabolites were examined using an untargeted, discovery-based approach (LC–MS) that includes more classes of metabolites other than lipids and amino acids for analyses, thus providing a more comprehensive metabolic profiling. Models with identified metabolites yielded higher indicative values on postpartum AGM than model using traditional risk factors and/or glycemic levels collected at index pregnancy. Such findings might suggest a great potential of utilizing these identified metabolites to underlie the transition between GDM and AGM. Two out of the five identified AGM-associated metabolites among women with a history of GDM were lysoPCs [lysoPC(16:1) and lysoPC(20:3)], both increased in women with AGM. LysoPCs are essential elements of glycerophospholipid metabolism and also reservoirs and transporters for fatty acids and choline^14^. Insulin resistance, prediabetes, and T2D are accompanied by hypertriglyceridemia^15^ and abnormal glycerophospholipid metabolism^16^. Previous studies have reported an upregulation of lysoPCs including lysoPC (16: 1) in women with GDM^16,17^. Therefore, the increase of lysoPCs found in our study might lead to a surplus in fatty acids and choline, which ultimately results in a higher risk of impairment in glucose metabolism^18^.

We also observed linoleic acid (LA) signatures, alpha-linoleic acid (ALA), and glycocholic acid in women with postpartum AGM. LA is a polyunsaturated fatty acid (PUFA) and a type of omega-6 fatty acid associated with reduced risk of T2D and improved glucose tolerance in women after GDM^19,20^. Incorporating linoleic acid into phospholipids could alter membrane fluidity and further enhance insulin receptor activity^21^. ALA is an essential omega-3 fatty acid and a precursor of eicosapentaenoic acid (EPA) and docosahexaenoic acid (DHA). It has been reported to be an active agent reducing circulating free fatty acids (FFA) and increasing insulin sensitivity and may reduce the risk of T2D^22^. Moreover, ALA has also shown the potential of lowering the levels of HbA_1c_ and fasting blood glucose concentrations in diabetic patients^23^. In addition, glycocholic acid is an acyl glycine and a bile acid-glycine conjugate involved in fats' emulsification and part of the primary bile acid biosynthesis pathway (KEGG ID: hsa00120). As we know, bile acids are physiological detergents that facilitate excretion, absorption, and transport of fats and sterols in the intestine and liver. The inter-organ signaling and interplay between bile acids receptors and the gut microbiota have been suggested to underlie the pathophysiology of T2D^24,25^.

Interestingly, we also observed decreased level of a metabolite (*p-cresol* sulfate) underlying AGM that was not reported to be associated with GDM or AGM. The level of circulating 4-cresol in the host organism may reflect architectural alterations of the gut microbiota in obese and diabetic patients^26,27^. However, the pathophysiological mechanisms of p-cresol sulfate's role in AGM development remain unclear and require further investigation.

This study's strengths lie in the application of AGM as an early stage outcome, measurements evaluated via standardized protocols, and reliable quality control on metabolites examination. However, our study is not without limitations. First, the relatively small sample size might have restricted the study power to detect more potential metabolites signatures underlying AGM. In addition to the small sample size, we were unable to match ethnicity completely. However, considering that all mothers were of Southeast Asian origin and no significant difference was found across ethnicities between GDM and non-GDM controls, the genetic heterogeneity in our findings might not be substantive. Second, residual bias might exist such as glycated hemoglobin (HbA1C) at index pregnancy. Third, we did not collect any dietary intake data after delivery. Even though some evidence showed no difference between the GDM and non-GDM group after delivery in terms of diet or energy intake^9,11^, further studies are warranted to include such variable. Fourth, since levels of the metabolites were examined at follow-up rather than at baseline, reverse causality cannot be ruled out in our preliminary findings. Future studies with larger sample sizes in a multiracial and prospective study setting with external validation, as well as multiple time points of metabolites testing, are warranted to verify these preliminary findings.

## Conclusion

Our study identified five metabolites including *p-cresol* sulfate, linoleic acid, glycocholic acid, lysoPC(16:1) and lysoPC(20:3) that were associated with postpartum AGM, specifically among women with prior GDM, beyond traditional risk factors. These metabolic signatures might shed light on the pathophysiology underlying the transition from GDM to AGM, and even provide insights into potential screening approaches using metabolites in clinical practice.

## Methods

### Study participants and design

This is a cross-sectional and observational pilot study nested in a longitudinal birth cohort study in Singapore (Growing Up in Singapore Towards Healthy Outcomes, GUSTO). This cohort recruited 1136 mothers with singleton pregnancies during their first trimester from June 2009 to September 2010. We have reported the study design and recruitment criteria in previous publications^28^. We performed an oral glucose tolerance test (OGTT) at 26–28 weeks of gestation for all recruited mothers. As a pilot in the metabolomics study, we enrolled a total of 100 participants in the current study, including 50 GDM and 50 non-GDM women matched for age (± 2 years), ethnicity and pre-pregnancy BMI (± 2 kg/m^2^ and within the same WHO category). All participants attended both baseline (26–28 weeks’ gestation) and follow-up (5-year postpartum) visits. Supplementary Fig. 1 presents the flowchart of the current study design.

### GDM diagnosis at the index pregnancy

At baseline, we diagnosed 50 women with GDM using a 2-h 75 g oral glucose tolerance test (OGTT) during 24–28 weeks gestation according to World Health Organization 1999 criteria^29^: fasting glucose ≥ 7.0 mmol/l and/or 2-h plasma glucose ≥ 7.8 mmol/l. None of these 50 women with GDM required drug treatment.

### Diagnosis of abnormal glucose metabolism (AGM) at 5-year postpartum

At the 5-year postpartum visit, we assessed glucose tolerance of all 100 participants using HbA_1c_ and a 2-h 75 g OGTT. We defined prediabetes as follows: (a) fasting plasma glucose 6.1‒6.9 mmol/l and 2-h plasma glucose < 11.0 mmol/lL, or (b) fasting plasma glucose < 7.0 mmol/l and 2-h plasma glucose 7.9‒11.0 mmol/l. We defined T2D as: (a) fasting plasma glucose ≥ 7.0 mmol/l, or (b) 2-h plasma glucose ≥ 11.0 mmol/l, or (c) HbA_1c_ ≥ 6.5%, or (d) self-reported physician-diagnosed T2D during the 5 years follow-up. We subsequently categorized participants as having AGM if they had either prediabetes or T2D at the 5-year postpartum visit.

### Liquid chromatograph–mass spectrometer (LC–MS) based metabolic profiling at 5-year postpartum follow-up

We extracted the metabolites using 200 μl serum samples collected at the 5-year postpartum visit. Briefly, we added 800 μl ice-cold mixture of methanol/acetone/acetonitrile (1:1:1, v/v/v) to each serum sample and incubated the mixture at − 20 °C for 30 min to precipitate. We then centrifuged the mixture at 16,000×*g* for 15 min (4 °C) to collect supernatant containing metabolites and dried the supernatant in a vacuum concentrator (miVac, GeneVac, Warminster, UK) before LC–MS analysis. We described the detailed laboratory procedures in Appendix, Supplementary Table 4 and Supplementary Figs. 2 and 3. We tested all samples in one batch.

We obtained and imported raw data from LC–MS analysis to MarkerView (SCIEX, Foster, California, US) for peak extraction, the lists of which contained m/z values, retention time and integrated ion intensity for each m/z feature. We employed a modified 80% rule for missing value handling, i.e., a metabolite feature is kept if the metabolite feature has a non-zero value for at least 80% in any group samples^30^. We applied interquartile range (IQR) to the peak lists and performed data filtering using *MetaboAnalyst (Version 4.0)*^31^. We filtered the data further if the relative standard deviation (RSD) were more than 20% in QC samples.

We detected a total of 21,226 metabolite features using LC–MS (1734 features from HILIC negative mode, 2636 features from HILIC positive mode, 6181 features from RP negative mode and 10,655 featured from RP positive) after applying the modified 80% rule. We finally included a total of 3067 metabolite features for statistical analysis after *MetaboAnalyst* processing.

### Covariates

We measured standing height using the SECA model 213 (Seca, Hamburg, Germany) and standing weight using SECA model 803 scale (Seca, Hamburg, Germany), according to standardized protocols^32^ at baseline and the 5-year follow-up visit. We calculated BMI as weight in kilogram over the square of height in meter. We calculated 26–28 weeks’ gestational weight gain (GWG) as the difference in weight between 26 and 28 weeks’ gestation and pre-pregnancy. Trained staff administered questionnaires in either English, Chinese, Malay, or Tamil at baseline index pregnancy. We collected information on the highest education level (college vs. below college), family history of diabetes (yes vs. no), past pregnancy history (parity, past GDM), and pre-pregnancy weight.

### Statistical analyses

#### Identifying candidate metabolites associated with 5-year postpartum AGM

First, we compared GDM and non-GDM women characteristics with generalized linear mixed models or generalized estimating equations when appropriate to account for matching factors. We constructed orthogonal projections to latent structures discriminant analysis (OPLS-DA) to separate and discriminate women with AGM and normal glucose metabolism. Generally, OPLS-DA aimed to differentiate between groups in highly complex datasets (e.g. LC–MS based metabolic data), despite within-group variability^33^. Second, we used variable importance for the projection (VIP) plot to summarize the importance of the metabolite features to the OPLS-DA model (VIP score > 1). Then we used univariate analysis (Mann–Whitney *U* test and t-test when appropriate, *p* < 0.05) to determine whether a metabolite showed different level between women with AGM and normal glucose metabolism. Third, we included these candidate metabolites for multivariable analysis.

#### Differentiating candidate metabolites associated with 5-year postpartum AGM between women with or without GDM at the index pregnancy

We stratified the participants based on their GDM status diagnosed at index pregnancy, and then used multivariable logistic regression models to identify metabolites specifically associated with 5-year postpartum AGM among women with a history of GDM. We employed false discovery rate (FDR) with the Benjamini–Hochberg procedure to correct for multiple testing and deemed significance at FDR less than 0.1. Next, we applied the ridge regression model to account for collinearity. We only included the metabolites with *p*-value < 0.05 in the ridge regression to further analyses.

#### Exploring the AUC in the regression model for 5-year postpartum AGM with serum metabolites among women with a history of GDM

We narrowed down the significant metabolites and used multivariable logistic regression models to assess their individual and combined indicative values on 5-year postpartum AGM. Using receiver operating characteristic (ROC) curves with tenfold cross-validation, we tested the following models: Model 1—known risk factors of postpartum AGM including age at follow-up, ethnicity, BMI at follow-up, family history of T2D, and the cumulative number of GDM episodes among all live pregnancies at follow-up; Model 2—Model 1, and additional baseline glucose parameters (fasting and 2-h glycemic levels at study entry); Models N—Model 2 and additional significant metabolites identified in our study, using a stepwise approach [e.g., each metabolite, each pair (if applicable), each triplet (if applicable), each quartet (if applicable), all metabolites (if applicable)]. We ranked the best fitting model with the highest R^2^ and area under curve (AUC) values. We further verified metabolites in the MS/MS spectrum's final model using pure chemical standards if commercial standards were available. The complete procedures of data processing and statistical analysis to discover metabolite features and identify candidate metabolites are illustrated in Supplementary Fig. 4.

#### Pathway analysis

We performed pathway analysis based on the KEGG (Kyoto Encyclopedia of Genes and Genomes) pathway database using *MetaboAnalyst* (*Version 4.0*). Such step aimed to investigate the published biological function in our significant metabolites identified in the OPLS-DA and univariate analysis. We also plotted a metabolic map (Fig. 3) to show the identified metabolic network.

We expressed data as median (interquartile range, IQR) or mean (standard deviation, SD) when appropriate. We conducted all statistical analyses using SIMCA 13.2 (Umetrics, Umea, Sweden), *MetaboAnalyst (Version 4.0),* and *R* Software *(Version 3.5.0)*, and deemed significance at *p*-value (2-sided) less than 0.05.

### Ethics approval

We conducted the study according to the tenets of the Declaration of Helsinki and obtained approval by the SingHealth Centralized Institutional Review Board and the National Health Group’s Domain Specific Review Board of Singapore. We obtained written informed consent from all participants for our study before any testing.

## Supplementary Information

Supplementary Legends.

Supplementary Methods.

Supplementary Figure 1.

Supplementary Figure 2.

Supplementary Figure 3.

Supplementary Figure 4.

Supplementary Tables.

## Data Availability

All datasets generated during and/or analyzed during the current study are not publicly available but are available from the corresponding author on reasonable request.

## References

[1] Proceedings of the 4th International Workshop-Conference on Gestational Diabetes Mellitus. Chicago, Illinois, USA, 14–16 March 1997. *Diabetes Care* **21**(Suppl 2), 1–167 (1998). 9841138

[2] McIntyre HD (2019). Gestational diabetes mellitus. Nat. Rev. Dis. Primers.

[3] Bellamy L, Casas JP, Hingorani AD, Williams D (2009). Type 2 diabetes mellitus after gestational diabetes: A systematic review and meta-analysis. Lancet.

[4] Li L-J (2018). Effect of gestational diabetes and hypertensive disorders of pregnancy on postpartum cardiometabolic risk. Endocr. Connect..

[5] Shah BR, Retnakaran R, Booth GL (2008). Increased risk of cardiovascular disease in young women following gestational diabetes mellitus. Diabetes Care.

[6] Beharier O (2015). Gestational diabetes mellitus is a significant risk factor for long-term maternal renal disease. J. Clin. Endocrinol. Metab..

[7] Fiehn O (2002). Metabolomics–the link between genotypes and phenotypes. Plant Mol Biol.

[8] Allalou A (2016). A predictive metabolic signature for the transition from gestational diabetes mellitus to type 2 diabetes. Diabetes.

[9] Khan SR (2019). The discovery of novel predictive biomarkers and early-stage pathophysiology for the transition from gestational diabetes to type 2 diabetes. Diabetologia.

[10] Lappas M (2015). The prediction of type 2 diabetes in women with previous gestational diabetes mellitus using lipidomics. Diabetologia.

[11] Tobias DK (2018). Dietary intakes and circulating concentrations of branched-chain amino acids in relation to incident type 2 diabetes risk among high-risk women with a history of gestational diabetes mellitus. Clin. Chem..

[12] Nanditha A (2016). Diabetes in Asia and the Pacific: Implications for the global epidemic. Diabetes Care.

[13] Guasch-Ferré M (2016). Metabolomics in prediabetes and diabetes: A systematic review and meta-analysis. Diabetes Care.

[14] Schmitz G, Ruebsaamen K (2010). Metabolism and atherogenic disease association of lysophosphatidylcholine. Atherosclerosis.

[15] Zhang X (2009). Human serum metabonomic analysis reveals progression axes for glucose intolerance and insulin resistance statuses. J. Proteome Res..

[16] Liu T (2016). Comprehensive analysis of serum metabolites in gestational diabetes mellitus by UPLC/Q-TOF-MS. Anal. Bioanal. Chem..

[17] Dudzik D (2014). Metabolic fingerprint of gestational diabetes mellitus. J. Proteomics.

[18] Svingen GF (2016). Prospective associations of systemic and urinary choline metabolites with incident type 2 diabetes. Clin. Chem..

[19] Wu JHY (2017). Omega-6 fatty acid biomarkers and incident type 2 diabetes: Pooled analysis of individual-level data for 39 740 adults from 20 prospective cohort studies. Lancet Diabetes Endocrinol..

[20] Andersson-Hall U, Carlsson NG, Sandberg AS, Holmang A (2018). Circulating linoleic acid is associated with improved glucose tolerance in women after gestational diabetes. Nutrients.

[21] Kröger J (2015). Erythrocyte membrane fatty acid fluidity and risk of type 2 diabetes in the EPIC-Potsdam study. Diabetologia.

[22] Barre DE (2007). The role of consumption of alpha-linolenic, eicosapentaenoic and docosahexaenoic acids in human metabolic syndrome and type 2 diabetes: A mini-review. J. Oleo Sci..

[23] Jovanovski E (2017). The effect of alpha-linolenic acid on glycemic control in individuals with type 2 diabetes: A systematic review and meta-analysis of randomized controlled clinical trials. Medicine.

[24] Jia W, Xie G, Jia W (2018). Bile acid-microbiota crosstalk in gastrointestinal inflammation and carcinogenesis. Nat. Rev. Gastroenterol. Hepatol..

[25] Chávez-Talavera O, Tailleux A, Lefebvre P, Staels B (2017). Bile acid control of metabolism and inflammation in obesity, type 2 diabetes, dyslipidemia, and nonalcoholic fatty liver disease. Gastroenterology.

[26] Meijers BK (2008). Free p-cresol is associated with cardiovascular disease in hemodialysis patients. Kidney Int..

[27] Meijers BK (2010). p-Cresol and cardiovascular risk in mild-to-moderate kidney disease. Clin. J. Am. Soc. Nephrol..

[28] Soh S-E (2014). Cohort profile: Growing up in Singapore towards healthy outcomes (GUSTO) birth cohort study. Int. J. Epidemiol..

[29] WHO. *WHO document Production Services* 19–20 (1999).

[30] Yang J, Zhao X, Lu X, Lin X, Xu G (2015). A data preprocessing strategy for metabolomics to reduce the mask effect in data analysis. Front. Mol. Biosci..

[31] Chong J (2018). MetaboAnalyst 4.0: Towards more transparent and integrative metabolomics analysis. Nucleic Acids Res..

[32] Li L-J (2012). Effect of maternal body mass index on the retinal microvasculature in pregnancy. Obstet. Gynecol..

[33] Bradley W, Robert P (2013). Multivariate analysis in metabolomics. Curr. Metab..

