# Supplementary material for: Metabolic signatures in the conversion from gestational diabetes mellitus to postpartum abnormal glucose metabolism: a pilot study in Asian women
Source: Sci Rep. 2021 Aug 12;11:16435. doi: 10.1038/s41598-021-95903-w (PMC8361021; doi:10.1038/s41598-021-95903-w)
Supplement: Supplementary file 1 — Supplementary Legends. [file 41598_2021_95903_MOESM1_ESM.pdf]

Supplementary Information Legends

Appendix

Supplementary Methods.

Supplementary Tables

Supplementary Table 1. Coefficients of Metabolites that were associated with 5-year postpartum abnormal glucose metabolism after multiple adjustment.

Supplementary Table 2. Ridge regression for candidate metabolites.

Supplementary Table 3. Performance of other candidate models for AGM at year 5.

Supplementary Table 4. ACCUITY LC parameter settings.

Supplementary Figures

Supplementary Figure 1. Study design flowchart.

Supplementary Figures 2. MS/MS spectrum of *p-cresol* sulfate from metabolomics analysis (top) verified with pure chemical standard (bottom).

Supplementary Figure 3. MS/MS spectrum of linoleic acid from metabolomics analysis (top) verified with pure chemical standard (bottom).

Supplementary Figure 4. The complete procedures illustrate the data processing and statistical analysis used for metabolite features discovery and candidate metabolites identification.
