# Supplementary material for: Metabolic signatures in the conversion from gestational diabetes mellitus to postpartum abnormal glucose metabolism: a pilot study in Asian women
Source: Sci Rep. 2021 Aug 12;11:16435. doi: 10.1038/s41598-021-95903-w (PMC8361021; doi:10.1038/s41598-021-95903-w)
Supplement: Supplementary file 2 — Supplementary Methods. [file 41598_2021_95903_MOESM2_ESM.docx]

**Appendix**

We analyzed each sample on reverse phase (RP) column and hydrophilic interaction chromatography (HILIC) column in positive and negative ionization modes, i.e. RP+, RP-, HILIC+, and HILIC- (**Supplementary Table S1**). We reconstituted aliquots for RP injections in 25 μl 2% acetonitrile and reconstituted aliquots for HILIC injections in 25 μl 80% acetonitrile, respectively. We performed metabolite separation on an ACQUITY I-class UPLC system (Waters, Milford, Massachusetts, US). The injection volume was 10 μl and the flow rate was 0.6 ml/min. We maintained the column and auto-sampler at 40 °C and 10 °C, respectively. We have listed columns, mobile phases and gradient settings for RP and HILIC separation in **Supplementary Table S1**. We prepared quality control (QC) samples by pooling equal volume of all serum samples in this study, in order to monitor the stability and repeatability during LC-MS analysis. The pretreatment of QC samples was the same as that of real samples. We injected the QC samples after every 10 real samples.

We achieved mass detection on a TripleTOF 5600 fitted with a DuoSpray ion source (SCIEX, Foster, California, US), and performed automatic mass calibration after every 20 injections using the automated calibration delivery system. We set the source voltage to 5500 V for positive ionization and -4500 V for negative ionization, of which the declustering potential was 80 V and the source temperature was 500 °C for both polarities. We also set the curtain gas flow, nebulizer and heater at 30, 55 and 60 arbitrary units, accordingly. We used information dependent acquisition (IDA) to collect full scan mass spectrometry (MS) and tandem mass spectrometry (MS/MS) information simultaneously, with a mass to charge ratio (m/z) range of 100 to 1000. The instrument performed a TOFMS survey with 160 ms accumulation time, followed by 5 MS/MS scans with 18 ms accumulation time. We ramped the collision energy linearly from 20 to 40 V and applied the following parameters to data acquisition: dynamic background subtraction, charger monitoring to exclude multiple charged ions, and dynamic exclusion of former target ions for 1s. In order to illustrate further, we displayed two plots on MS/MS spectrum of *p-cresol* sulfate and linoleic acid verified with pure chemical standard **Supplementary Figure S1 and S2**, respectively.
