# Supplementary figures and images for: Metabolic signatures in the conversion from gestational diabetes mellitus to postpartum abnormal glucose metabolism: a pilot study in Asian women
Source: Sci Rep. 2021 Aug 12;11:16435. doi: 10.1038/s41598-021-95903-w (PMC8361021; doi:10.1038/s41598-021-95903-w)

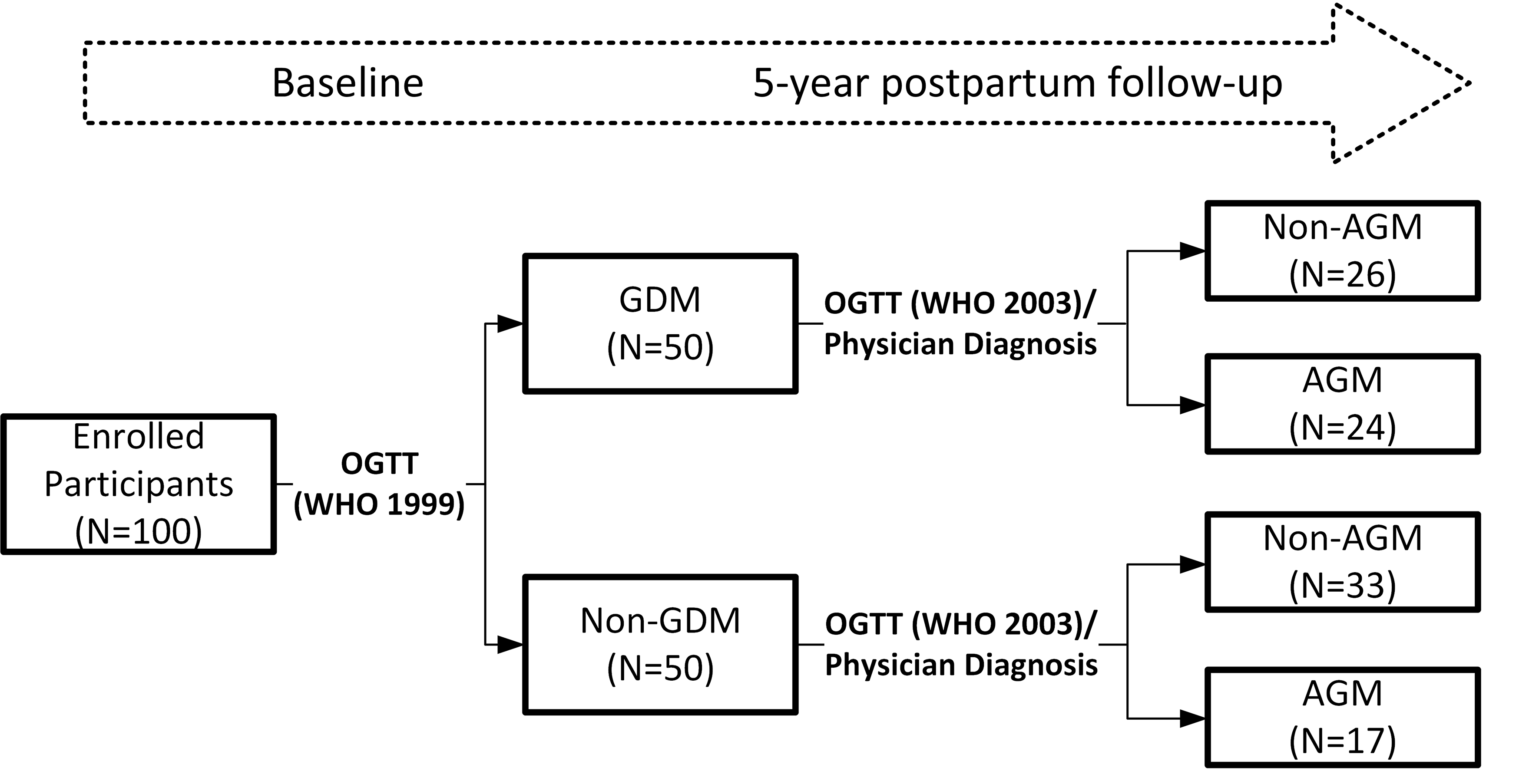

Supplement: Supplementary file 3 — Supplementary Figure 1. [file 41598_2021_95903_MOESM3_ESM.tif]

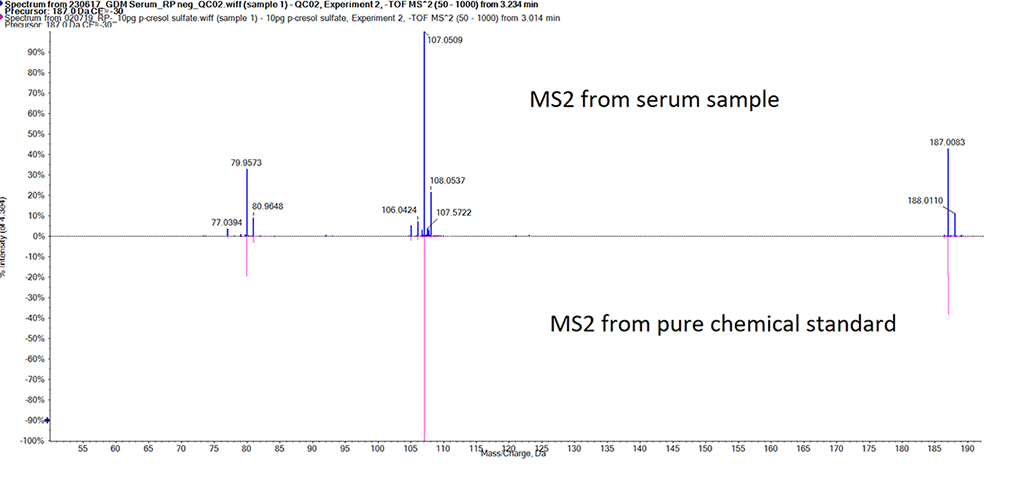

Supplement: Supplementary file 4 — Supplementary Figure 2. [file 41598_2021_95903_MOESM4_ESM.tif]

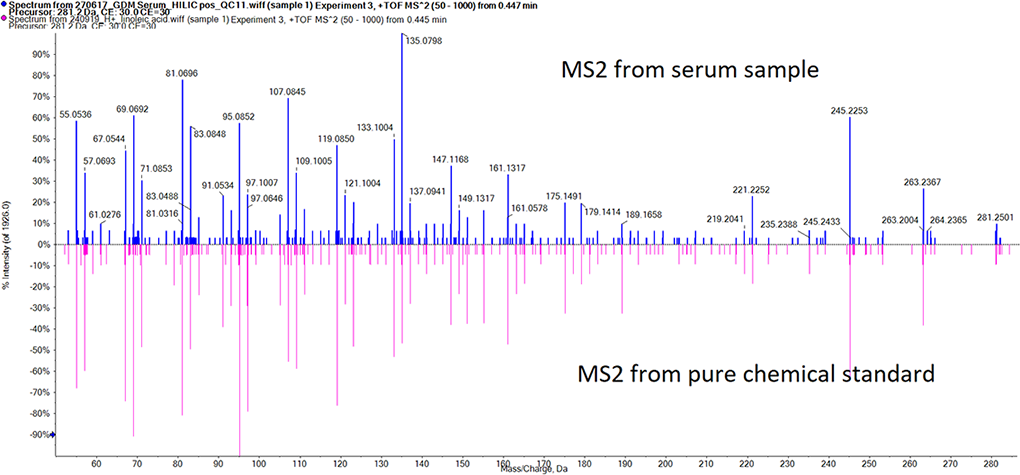

Supplement: Supplementary file 5 — Supplementary Figure 3. [file 41598_2021_95903_MOESM5_ESM.tif]

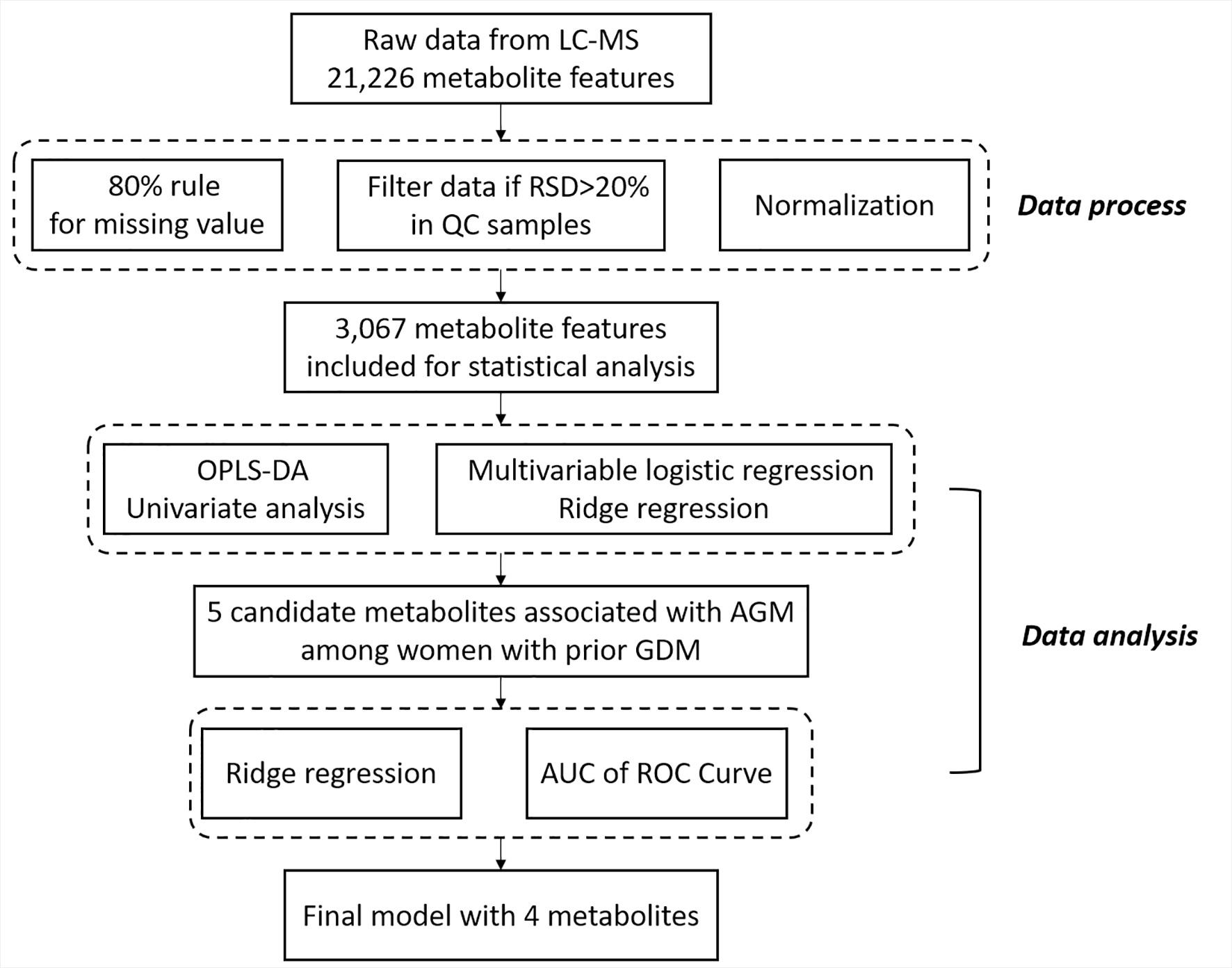

Supplement: Supplementary file 6 — Supplementary Figure 4. [file 41598_2021_95903_MOESM6_ESM.tif]
