# Supplementary material for: Metabolic signatures in the conversion from gestational diabetes mellitus to postpartum abnormal glucose metabolism: a pilot study in Asian women
Source: Sci Rep. 2021 Aug 12;11:16435. doi: 10.1038/s41598-021-95903-w (PMC8361021; doi:10.1038/s41598-021-95903-w)
Supplement: Supplementary file 7 — Supplementary Tables. [file 41598_2021_95903_MOESM7_ESM.docx]

**Supplementary Table 1. Coefficients of Metabolites that were associated with 5-year postpartum abnormal glucose metabolism after multiple adjustment**

| **Metabolite ID** | **All participants regardless of baseline GDM** | | | | | **Women with GDM at baseline** | | | | | **Women with normal glucose metabolism at baseline** | | | | |
| --- | --- | --- | --- | --- | --- | --- | --- | --- | --- | --- | --- | --- | --- | --- | --- |
|  | β | 95CI  low | 95CI  up | *p-*value | FDR | β | 95CI  low | 95CI  up | *p*-value | FDR | β | 95CI  low | 95CI  up | *p-*value | FDR |
| 187.0077_3.20_rn | -0.21 | -0.37 | -0.06 | 0.008 | 0.052 | -0.35 | -0.64 | -0.11 | 0.009 | 0.074 | -0.20 | -0.49 | 0.04 | 0.124 | 0.204 |
| 212.0026_2.41_rn | -0.67 | -1.33 | -0.10 | 0.030 | 0.085 | -0.71 | -1.61 | 0.06 | 0.087 | 0.133 | -1.05 | -2.65 | 0.16 | 0.135 | 0.204 |
| 217.0177_3.95_rn | -0.67 | -1.33 | -0.10 | 0.030 | 0.085 | -0.71 | -1.61 | 0.06 | 0.087 | 0.133 | -1.05 | -2.65 | 0.16 | 0.135 | 0.204 |
| 254.9827_0.42_rn | -8.61 | -15.19 | -3.12 | 0.005 | 0.052 | -8.05 | -18.36 | 0.37 | 0.085 | 0.133 | -13.15 | -29.71 | -2.32 | 0.057 | 0.186 |
| 271.2053_6.85_rp | 5.47 | 0.90 | 10.34 | 0.022 | 0.077 | 6.05 | -1.33 | 14.29 | 0.120 | 0.153 | 5.99 | -0.77 | 13.70 | 0.096 | 0.204 |
| 274.1047_0.42_rn | -9.39 | -17.08 | -2.47 | 0.011 | 0.058 | -3.19 | -14.48 | 7.51 | 0.563 | 0.563 | -20.08 | -37.30 | -6.89 | 0.008 | 0.068 |
| 279.2316_0.44_hp | -4.61 | -8.38 | -1.45 | 0.008 | 0.052 | -8.20 | -16.84 | -1.51 | 0.032 | 0.102 | -4.09 | -9.31 | 0.12 | 0.080 | 0.204 |
| 279.2323_9.57_rp | 2.94 | 0.73 | 5.44 | 0.013 | 0.060 | 3.29 | -0.28 | 7.88 | 0.103 | 0.144 | 2.39 | -0.89 | 6.10 | 0.169 | 0.216 |
| 281.2476_0.44_hp | -3.58 | -6.46 | -1.14 | 0.008 | 0.052 | -7.96 | -16.26 | -2.24 | 0.023 | 0.087 | -2.06 | -6.04 | 1.20 | 0.251 | 0.304 |
| 351.1640_4.20_rn | 23.35 | 9.09 | 39.73 | 0.003 | 0.052 | 23.37 | 2.87 | 49.34 | 0.043 | 0.125 | 32.63 | 6.42 | 67.31 | 0.031 | 0.140 |
| 367.1062_0.46_rn | -1.39 | -2.45 | -0.48 | 0.005 | 0.052 | -0.96 | -2.43 | 0.24 | 0.143 | 0.102 | -2.26 | -4.30 | -0.62 | 0.013 | 0.101 |
| 369.1742_5.47_rn | 0.13 | 0.02 | 0.24 | 0.022 | 0.077 | 0.17 | 0.01 | 0.37 | 0.056 | 0.169 | 0.09 | -0.07 | 0.27 | 0.275 | 0.316 |
| 369.1746_4.93_rn | 0.84 | 0.31 | 1.46 | 0.004 | 0.052 | 1.05 | 0.20 | 2.19 | 0.035 | 0.102 | 1.16 | 0.24 | 2.28 | 0.023 | 0.133 |
| 378.1011_5.22_rn | -5.89 | -11.50 | -1.56 | 0.020 | 0.077 | -4.94 | -13.80 | 0.67 | 0.183 | 0.201 | -7.47 | -18.54 | 0.21 | 0.116 | 0.204 |
| 429.3001_8.36_rp | 8.52 | 2.68 | 15.30 | 0.007 | 0.052 | 8.26 | -2.92 | 23.03 | 0.204 | 0.213 | 16.12 | 6.01 | 30.21 | 0.007 | 0.068 |
| 446.2915_5.37_rn | -1.80 | -6.50 | 2.02 | 0.391 | 0.537 | -16.63 | -33.81 | -5.17 | 0.019 | 0.074 | 4.44 | -1.13 | 10.35 | 0.111 | 0.204 |
| 449.1307_4.15_rn | 4.37 | 1.74 | 7.56 | 0.003 | 0.052 | 4.43 | 0.74 | 9.59 | 0.042 | 0.125 | 6.45 | 1.26 | 13.62 | 0.036 | 0.140 |
| 464.3024_4.78_rn | -6.28 | -12.32 | -1.76 | 0.022 | 0.077 | -9.96 | -23.93 | -1.63 | 0.107 | 0.144 | -6.25 | -16.14 | 0.95 | 0.150 | 0.204 |
| 471.2434_4.21_rn | 5.13 | 1.28 | 10.63 | 0.029 | 0.085 | 6.07 | 0.88 | 16.09 | 0.076 | 0.133 | 3.68 | -3.05 | 13.07 | 0.336 | 0.368 |
| 494.3249_8.41_rp | 0.28 | -0.01 | 0.59 | 0.069 | 0.150 | 1.13 | 0.29 | 2.26 | 0.023 | 0.087 | 0.10 | -0.28 | 0.50 | 0.585 | 0.585 |
| 546.3563_8.78_rp | 0.77 | 0.12 | 1.48 | 0.024 | 0.078 | 1.41 | 0.44 | 2.88 | 0.019 | 0.074 | 0.36 | -0.63 | 1.38 | 0.475 | 0.497 |
| 559.4713_0.44_hp | -4.05 | -7.51 | -1.09 | 0.012 | 0.060 | -8.07 | -17.57 | -1.24 | 0.046 | 0.125 | -3.20 | -8.08 | 0.87 | 0.151 | 0.204 |
| 577.4823_0.44_hp | -1.21 | -2.20 | -0.37 | 0.009 | 0.052 | -2.40 | -5.23 | -0.42 | 0.042 | 0.125 | -0.95 | -2.31 | 0.19 | 0.126 | 0.204 |

**Supplementary Table 2. Ridge regression for candidate metabolites.**

| **Independent Variables** | **Chemical Name** | **β** | **Standard Error (scaled)** | ***p*-value** |
| --- | --- | --- | --- | --- |
| 187.0077_3.20_rn | p-cresol sulfate | -0.001 | 0.017 | 0.001 |
| 281.2476_0.44_hp | Linoleic acid | -0.032 | 0.017 | 0.018 |
| 446.2915_5.37_rn | Glycocholic acid | -0.062 | 0.017 | 0.007 |
| 494.3249_8.41_rp | LysoPC(16:1) | 0.006 | 0.017 | 0.002 |
| 546.3563_8.78_rp | LysoPC(20:3) | 0.007 | 0.017 | 0.003 |

**Supplementary Table 3. Performance of other candidate models for AGM at year 5.**

| **Model ID** | **Included Metabolites** | **AUC** | **R^2^** |
| --- | --- | --- | --- |
| Model 187 | *p-cresol* sulfate | 0.804 | 0.366 |
| Model 281 | linoleic acid | 0.793 | 0.355 |
| Model 446 | Glycocholic acid | 0.818 | 0.406 |
| Model 494 | lysoPC(16:1) | 0.811 | 0.410 |
| Model 546 | lysoPC(20:3) | 0.819 | 0.386 |
| Model 187+281 | *p-cresol* sulfate + linoleic acid | 0.829 | 0.474 |
| Model 187+446 | *p-cresol* sulfate + Glycocholic acid | 0.829 | 0.443 |
| Model 187+494 | *p-cresol* sulfate + lysoPC(16:1) | 0.856 | 0.489 |
| Model 187+546 | *p-cresol* sulfate + lysoPC(20:3) | 0.836 | 0.432 |
| Model 281+446 | linoleic acid + Glycocholic acid | 0.851 | 0.478 |
| Model 281+494 | linoleic acid + lysoPC(16:1) | 0.866 | 0.540 |
| Model 281+546 | linoleic acid + lysoPC(20:3) | 0.875 | 0.498 |
| Model 446+494 | Glycocholic acid + lysoPC(16:1) | 0.873 | 0.537 |
| Model 446+546 | Glycocholic acid + lysoPC(20:3) | 0.930 | 0.672 |
| Model 187+281+446 | *p-cresol* sulfate + linoleic acid + Glycocholic acid | 0.868 | 0.525 |
| Model 187+281+494 | *p-cresol* sulfate + linoleic acid + lysoPC(16:1) | 0.906 | 0.620 |
| Model 187+281+546 | *p-cresol* sulfate + linoleic acid + lysoPC(20:3) | 0.891 | 0.553 |
| Model 187+446+494 | *p-cresol* sulfate + Glycocholic acid + lysoPC(16:1) | 0.883 | 0.560 |
| Model 187+446+546 | *p-cresol* sulfate + Glycocholic acid + lysoPC(20:3) | 0.857 | 0.490 |
| Model 281+446+494 | linoleic acid + Glycocholic acid + lysoPC(16:1) | 0.921 | 0.642 |
| Model 281+446+546 | linoleic acid + Glycocholic acid + lysoPC(20:3) | 0.893 | 0.573 |

**Supplementary Table 4. ACCUITY LC parameter settings**

| Analysis mode | Column | Mobile Phases | Gradient |
| --- | --- | --- | --- |
| RP+ | ACQUITY UPLC HSS T3 (2.1 x 100 mm, 1.8 μm) | 0.1% formic acid in water (A) and 0.1% formic acid in acetonitrile (B) | 0-1 min, 2% B; 1-4.5 min, 2%-20% B; 4.5-7 min, 20%-50% B; 7-9.5 min, 50%-95% B; 9.5-11 min, 95% B; 11-11.5 min, 95% to 2% B; 11.5-15 min, 2%B. |
| RP- | ACQUITY UPLC BEH C18 (2.1 x 100 mm, 1.7 μm) | 6.5 mM ammonium bicarbonate in water (A) and 6.5 mM ammonium bicarbonate in 95% acetonitrile (B) | 0-1 min, 2% B; 1-3.5 min, 2%-20% B; 3.5-9 min, 20%-100% B; 9-10.5 min, 100% B; 10.5-11 min, 100% to 2% B; 11-14 min, 2%B. |
| HILIC+ | ACQUITY UPLC BEH Amide (2.1 x 100 mm, 1.7 μm) | 10 mM ammonium formate + 0.05% formic acid in water (A) and 10 mM ammonium formate + 0.05% formic acid in 90% acetonitrile | 0-1 min, 95% B; 1-8 min, 95%-60% B; 8-8.5 min, 60%-20% B; 8.5-9.5 min, 20% B; 9.5-10 min, 20% to 95% B; 10-16 min, 95%B. |
| HILIC- | ACQUITY UPLC BEH HILIC (2.1 x 100 mm, 1.7 μm) | 6.5 mM ammonium bicarbonate in water (A) and 10 mM ammonium bicarbonate in 95% acetonitrile | 0-1 min, 95% B; 1-8 min, 95%-60% B; 8-8.5 min, 60%-20% B; 8.5-9.5 min, 20% B; 9.5-10 min, 20% to 95% B; 10-16 min, 95%B. |
